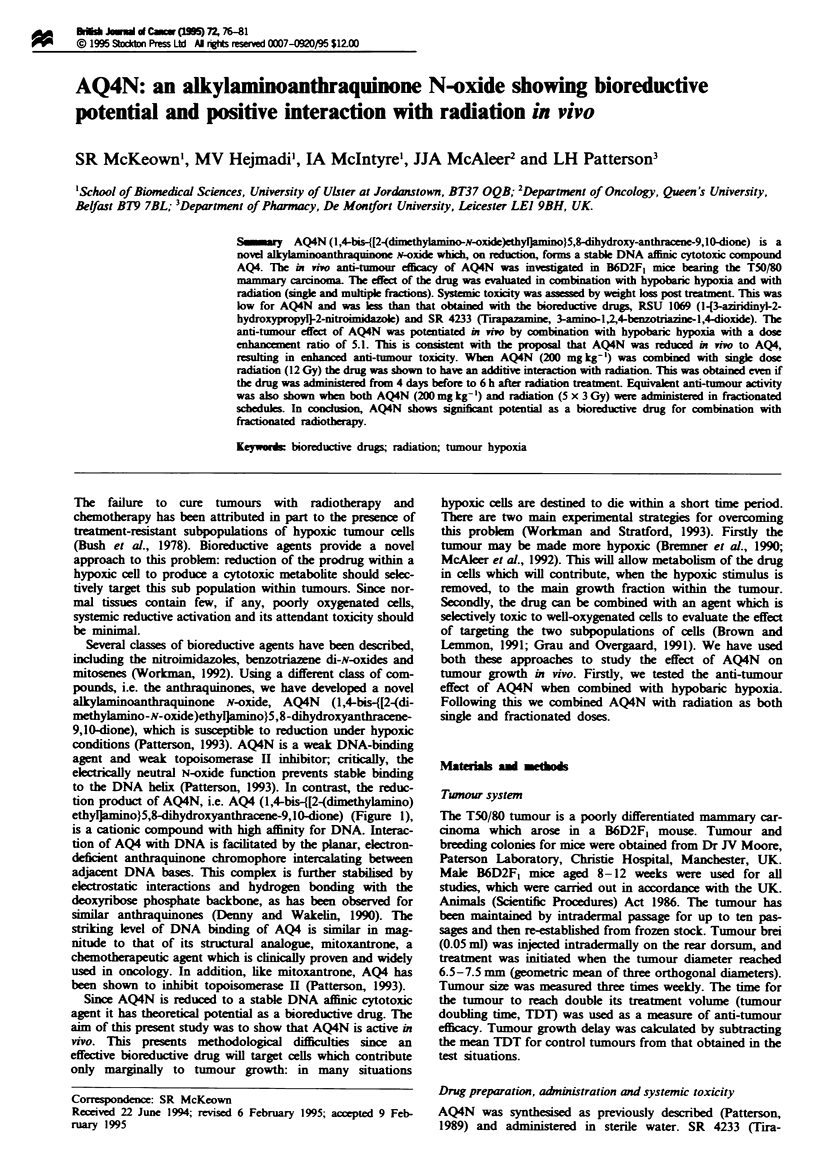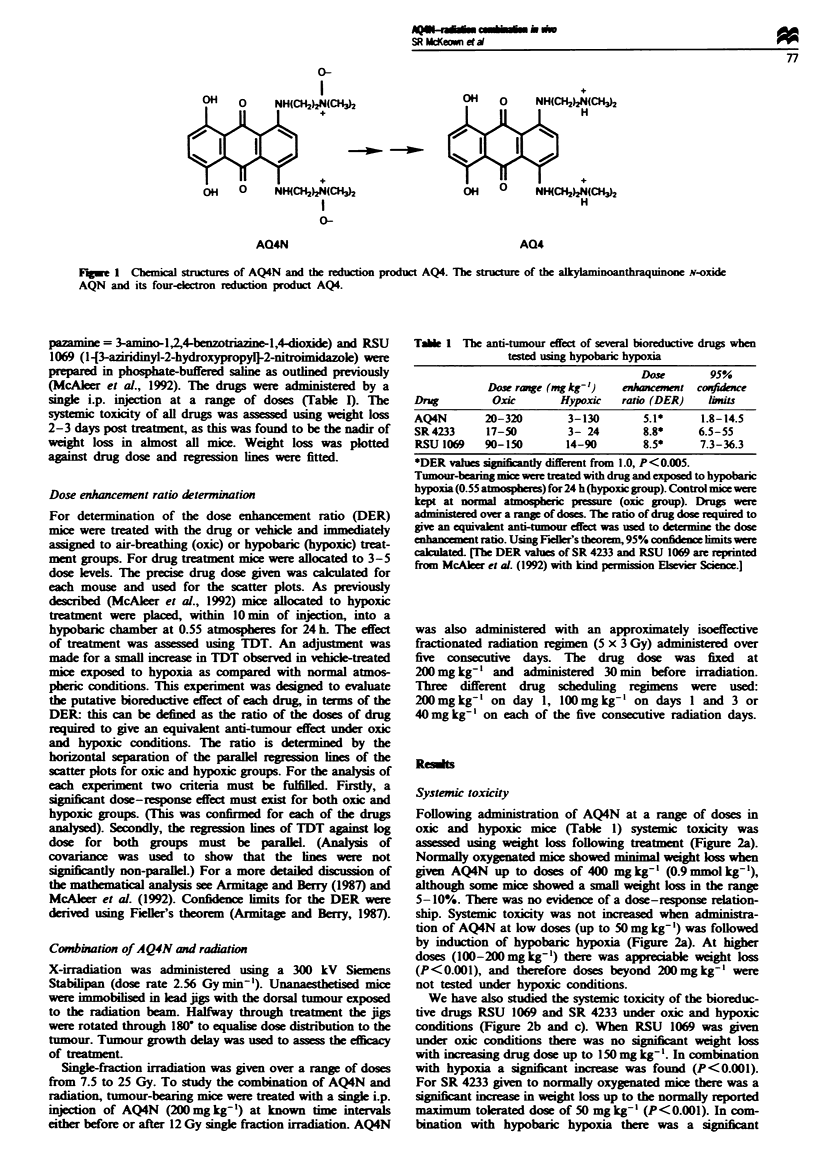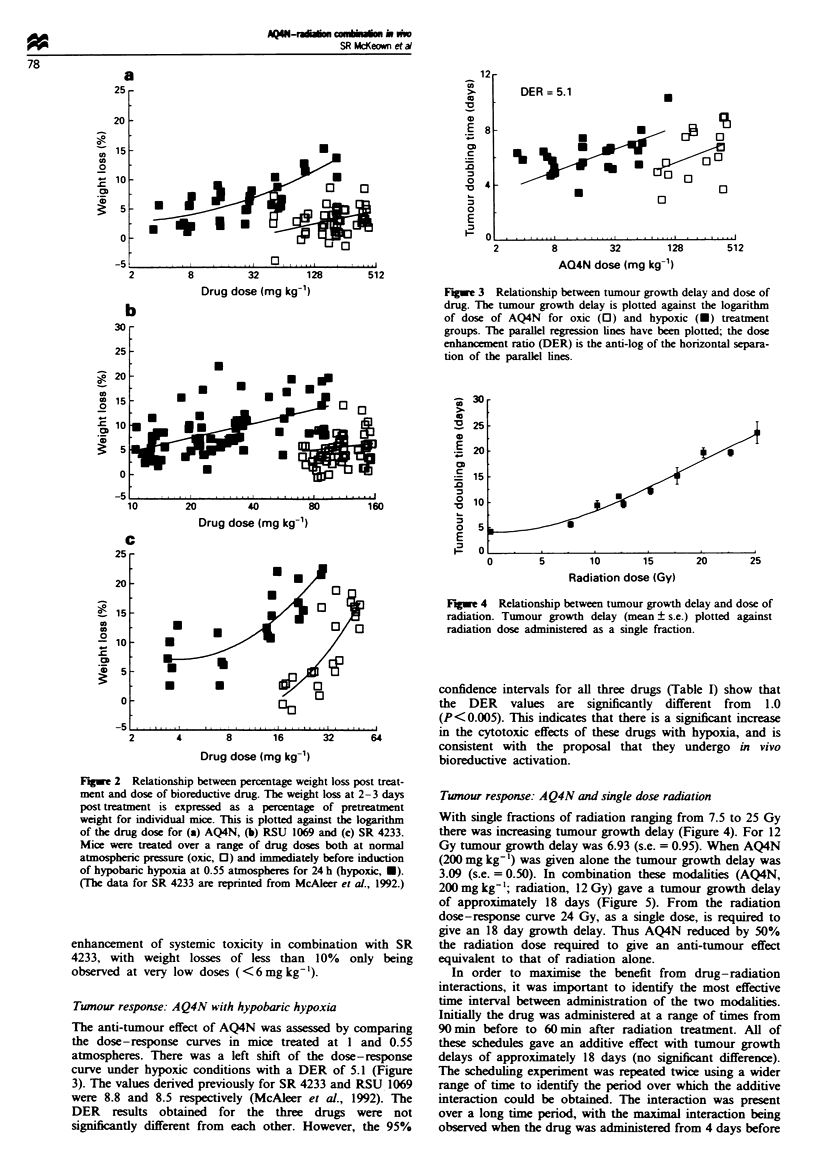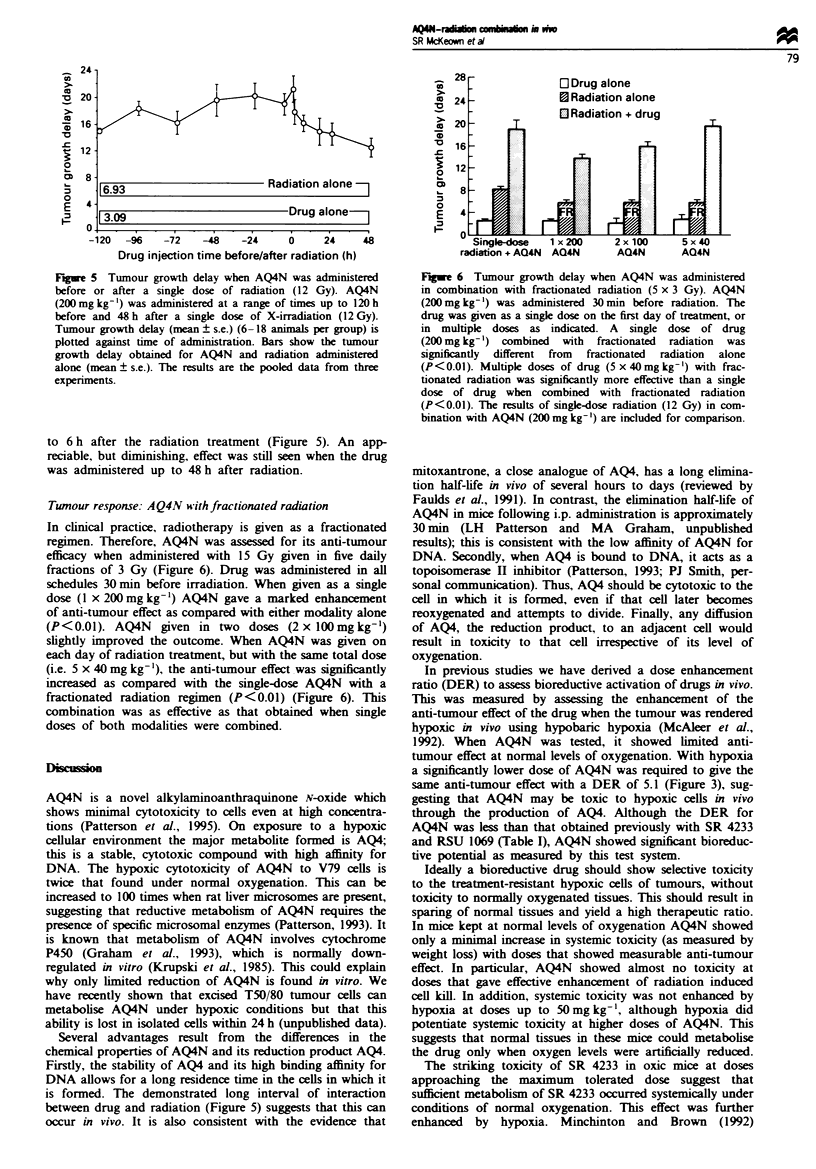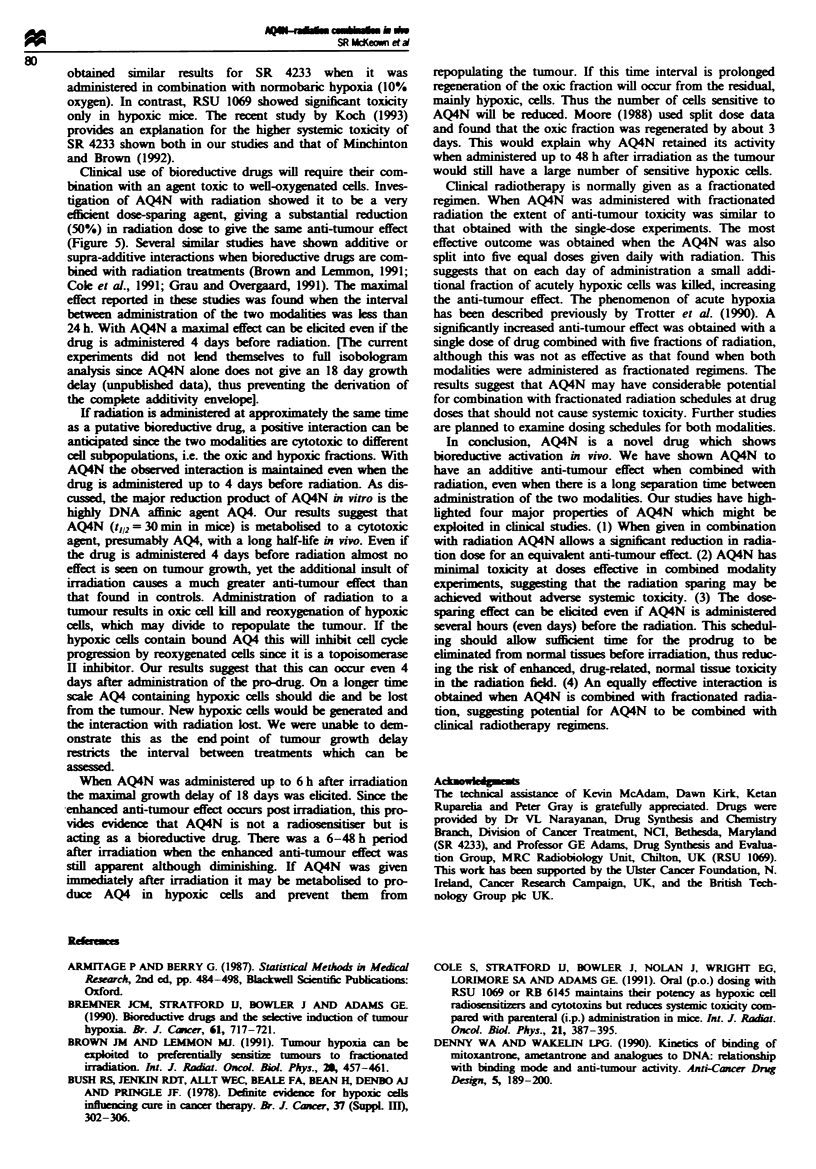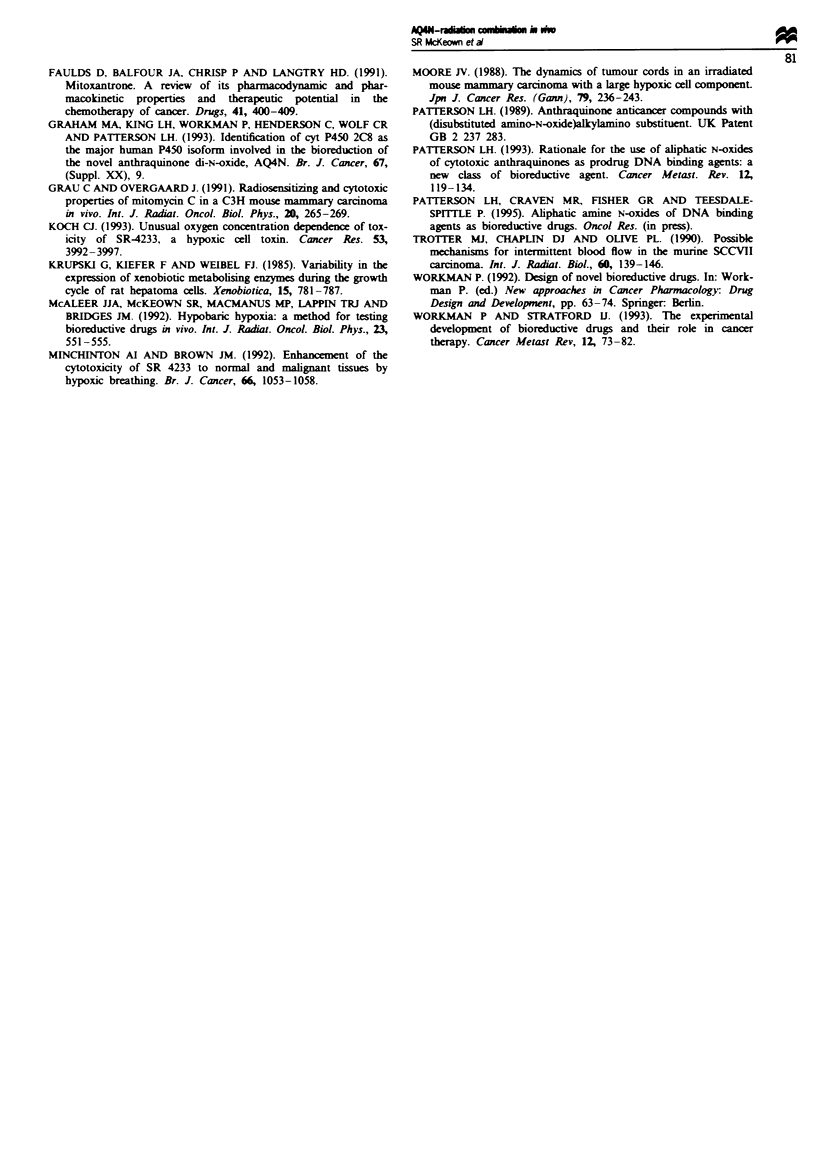# AQ4N: an alkylaminoanthraquinone N-oxide showing bioreductive potential and positive interaction with radiation in vivo.

**DOI:** 10.1038/bjc.1995.280

**Published:** 1995-07

**Authors:** S. R. McKeown, M. V. Hejmadi, I. A. McIntyre, J. J. McAleer, L. H. Patterson

**Affiliations:** School of Biomedical Sciences, University of Ulster at Jordanstown, UK.

## Abstract

AQ4N (1,4-bis([2-(dimethylamino-N-oxide)ethyl]amino)5,8-dihydroxy- anthracene-9,10-dione) is a novel alkylaminoanthraquinone N-oxide which, on reduction, forms a stable DNA affinic cytotoxic compound AQ4. The in vivo anti-tumour efficacy of AQ4N was investigated in B6D2F1 mice bearing the T50/80 mammary carcinoma. The effect of the drug was evaluated in combination with hypobaric hypoxia and with radiation (single and multiple fractions). Systemic toxicity was assessed by weight loss post treatment. This was low for AQ4N and was less than that obtained with the bioreductive drugs, RSU 1069 (1-[3-aziridinyl-2-hydroxypropyl]-2-nitroimidazole) and SR 4233 (Tirapazamine, 3-amino-1,2,4-benzotriazine-1,4-dioxide). The anti-tumour effect of AQ4N was potentiated in vivo by combination with hypobaric hypoxia with a dose enhancement ratio of 5.1. This is consistent with the proposal that AQ4N was reduced in vivo to AQ4, resulting in enhanced anti-tumour toxicity. When AQ4N (200 mg kg-1) was combined with single dose radiation (12 Gy) the drug was shown to have an additive interaction with radiation. This was obtained even if the drug was administered from 4 days before to 6 h after radiation treatment. Equivalent anti-tumour activity was also shown when both AQ4N (200 mg kg-1) and radiation (5 x 3 Gy) were administered in fractionated schedules. In conclusion, AQ4N shows significant potential as a bioreductive drug for combination with fractionated radiotherapy.


### AQ4N: an alkylaminoanthraquinone N-oxide showing bioreductive potential and positive interaction with radiation *in vivo*

SR McKeown[1], MV Hejmadi[1], IA McIntyre[1], JJA McAleer[2] and LH Patterson[3]

[1]*School of Biomedical Sciences, University of Ulster at Jordanstown, BT37 0QB;* [2]*Department of Oncology, Queen's University, Belfast BT9 7BL;* [3]*Department of Pharmacy, De Montfort University, Leicester LE1 9BH, UK.*

**Summary** AQ4N (1,4-bis-{[2-(dimethylamino-*N*-oxide)ethyl]amino}5,8-dihydroxy-anthracene-9,10-dione) is a novel alkylaminoanthraquinone *N*-oxide which, on reduction, forms a stable DNA affinic cytotoxic compound AQ4. The *in vivo* anti-tumour efficacy of AQ4N was investigated in $B6D2F_1$ mice bearing the T50/80 mammary carcinoma. The effect of the drug was evaluated in combination with hypobaric hypoxia and with radiation (single and multiple fractions). Systemic toxicity was assessed by weight loss post treatment. This was low for AQ4N and was less than that obtained with the bioreductive drugs, RSU 1069 (1-[3-aziridinyl-2-hydroxypropyl]-2-nitroimidazole) and SR 4233 (Tirapazamine, 3-amino-1,2,4-benzotriazine-1,4-dioxide). The anti-tumour effect of AQ4N was potentiated *in vivo* by combination with hypobaric hypoxia with a dose enhancement ratio of 5.1. This is consistent with the proposal that AQ4N was reduced *in vivo* to AQ4, resulting in enhanced anti-tumour toxicity. When AQ4N (200 mg $kg^{-1}$) was combined with single dose radiation (12 Gy) the drug was shown to have an additive interaction with radiation. This was obtained even if the drug was administered from 4 days before to 6 h after radiation treatment. Equivalent anti-tumour activity was also shown when both AQ4N (200 mg $kg^{-1}$) and radiation (5 × 3 Gy) were administered in fractionated schedules. In conclusion, AQ4N shows significant potential as a bioreductive drug for combination with fractionated radiotherapy.

**Keywords:** bioreductive drugs; radiation; tumour hypoxia

The failure to cure tumours with radiotherapy and chemotherapy has been attributed in part to the presence of treatment-resistant subpopulations of hypoxic tumour cells (Bush *et al.*, 1978). Bioreductive agents provide a novel approach to this problem: reduction of the prodrug within a hypoxic cell to produce a cytotoxic metabolite should selectively target this sub population within tumours. Since normal tissues contain few, if any, poorly oxygenated cells, systemic reductive activation and its attendant toxicity should be minimal.

Several classes of bioreductive agents have been described, including the nitroimidazoles, benzotriazene di-*N*-oxides and mitosenes (Workman, 1992). Using a different class of compounds, i.e. the anthraquinones, we have developed a novel alkylaminoanthraquinone *N*-oxide, AQ4N (1,4-bis-{[2-(dimethylamino-*N*-oxide)ethyl]amino}5,8-dihydroxyanthracene-9,10-dione), which is susceptible to reduction under hypoxic conditions (Patterson, 1993). AQ4N is a weak DNA-binding agent and weak topoisomerase II inhibitor; critically, the electrically neutral N-oxide function prevents stable binding to the DNA helix (Patterson, 1993). In contrast, the reduction product of AQ4N, i.e. AQ4 (1,4-bis-{[2-(dimethylamino) ethyl]amino}5,8-dihydroxyanthracene-9,10-dione) (Figure 1), is a cationic compound with high affinity for DNA. Interaction of AQ4 with DNA is facilitated by the planar, electron-deficient anthraquinone chromophore intercalating between adjacent DNA bases. This complex is further stabilised by electrostatic interactions and hydrogen bonding with the deoxyribose phosphate backbone, as has been observed for similar anthraquinones (Denny and Wakelin, 1990). The striking level of DNA binding of AQ4 is similar in magnitude to that of its structural analogue, mitoxantrone, a chemotherapeutic agent which is clinically proven and widely used in oncology. In addition, like mitoxantrone, AQ4 has been shown to inhibit topoisomerase II (Patterson, 1993).

Since AQ4N is reduced to a stable DNA affinic cytotoxic agent it has theoretical potential as a bioreductive drug. The aim of this present study was to show that AQ4N is active *in vivo*. This presents methodological difficulties since an effective bioreductive drug will target cells which contribute only marginally to tumour growth: in many situations hypoxic cells are destined to die within a short time period. There are two main experimental strategies for overcoming this problem (Workman and Stratford, 1993). Firstly the tumour may be made more hypoxic (Bremner *et al.*, 1990; McAleer *et al.*, 1992). This will allow metabolism of the drug in cells which will contribute, when the hypoxic stimulus is removed, to the main growth fraction within the tumour. Secondly, the drug can be combined with an agent which is selectively toxic to well-oxygenated cells to evaluate the effect of targeting the two subpopulations of cells (Brown and Lemmon, 1991; Grau and Overgaard, 1991). We have used both these approaches to study the effect of AQ4N on tumour growth *in vivo*. Firstly, we tested the anti-tumour effect of AQ4N when combined with hypobaric hypoxia. Following this we combined AQ4N with radiation as both single and fractionated doses.

### Materials and methods

### *Tumour system*

The T50/80 tumour is a poorly differentiated mammary carcinoma which arose in a $B6D2F_1$ mouse. Tumour and breeding colonies for mice were obtained from Dr JV Moore, Paterson Laboratory, Christie Hospital, Manchester, UK. Male $B6D2F_1$ mice aged 8–12 weeks were used for all studies, which were carried out in accordance with the UK. Animals (Scientific Procedures) Act 1986. The tumour has been maintained by intradermal passage for up to ten passages and then re-established from frozen stock. Tumour brei (0.05 ml) was injected intradermally on the rear dorsum, and treatment was initiated when the tumour diameter reached 6.5–7.5 mm (geometric mean of three orthogonal diameters). Tumour size was measured three times weekly. The time for the tumour to reach double its treatment volume (tumour doubling time, TDT) was used as a measure of anti-tumour efficacy. Tumour growth delay was calculated by subtracting the mean TDT for control tumours from that obtained in the test situations.

### *Drug preparation, administration and systemic toxicity*

AQ4N was synthesised as previously described (Patterson, 1989) and administered in sterile water. SR 4233 (Tira-

Correspondence: SR McKeown
Received 22 June 1994; revised 6 February 1995; accepted 9 February 1995

Figure 1 Chemical structures of AQ4N and the reduction product AQ4. The structure of the alkylaminoanthraquinone N-oxide AQN and its four-electron reduction product AQ4.

pazamine = 3-amino-1,2,4-benzotriazine-1,4-dioxide) and RSU 1069 (1-[3-aziridinyl-2-hydroxypropyl]-2-nitroimidazole) were prepared in phosphate-buffered saline as outlined previously (McAleer *et al.*, 1992). The drugs were administered by a single i.p. injection at a range of doses (Table I). The systemic toxicity of all drugs was assessed using weight loss 2–3 days post treatment, as this was found to be the nadir of weight loss in almost all mice. Weight loss was plotted against drug dose and regression lines were fitted.

*Dose enhancement ratio determination*

For determination of the dose enhancement ratio (DER) mice were treated with the drug or vehicle and immediately assigned to air-breathing (oxic) or hypobaric (hypoxic) treatment groups. For drug treatment mice were allocated to 3–5 dose levels. The precise drug dose given was calculated for each mouse and used for the scatter plots. As previously described (McAleer *et al.*, 1992) mice allocated to hypoxic treatment were placed, within 10 min of injection, into a hypobaric chamber at 0.55 atmospheres for 24 h. The effect of treatment was assessed using TDT. An adjustment was made for a small increase in TDT observed in vehicle-treated mice exposed to hypoxia as compared with normal atmospheric conditions. This experiment was designed to evaluate the putative bioreductive effect of each drug, in terms of the DER: this can be defined as the ratio of the doses of drug required to give an equivalent anti-tumour effect under oxic and hypoxic conditions. The ratio is determined by the horizontal separation of the parallel regression lines of the scatter plots for oxic and hypoxic groups. For the analysis of each experiment two criteria must be fulfilled. Firstly, a significant dose–response effect must exist for both oxic and hypoxic groups. (This was confirmed for each of the drugs analysed). Secondly, the regression lines of TDT against log dose for both groups must be parallel. (Analysis of covariance was used to show that the lines were not significantly non-parallel.) For a more detailed discussion of the mathematical analysis see Armitage and Berry (1987) and McAleer *et al.* (1992). Confidence limits for the DER were derived using Fieller's theorem (Armitage and Berry, 1987).

*Combination of AQ4N and radiation*

X-irradiation was administered using a 300 kV Siemens Stabilipan (dose rate 2.56 Gy $min^{-1}$). Unanaesthetised mice were immobilised in lead jigs with the dorsal tumour exposed to the radiation beam. Halfway through treatment the jigs were rotated through 180° to equalise dose distribution to the tumour. Tumour growth delay was used to assess the efficacy of treatment.

Single-fraction irradiation was given over a range of doses from 7.5 to 25 Gy. To study the combination of AQ4N and radiation, tumour-bearing mice were treated with a single i.p. injection of AQ4N (200 mg $kg^{-1}$) at known time intervals either before or after 12 Gy single fraction irradiation. AQ4N

**Table 1** The anti-tumour effect of several bioreductive drugs when tested using hypobaric hypoxia

| *Drug* | *Dose range (mg kg⁻¹)* Oxic | *Hypoxic* | *Dose enhancement ratio (DER)* | *95% confidence limits* |
|---|---|---|---|---|
| AQ4N | 20–320 | 3–130 | 5.1* | 1.8–14.5 |
| SR 4233 | 17–50 | 3– 24 | 8.8* | 6.5–55 |
| RSU 1069 | 90–150 | 14–90 | 8.5* | 7.3–36.3 |

*DER values significantly different from 1.0, $P<0.005$.
Tumour-bearing mice were treated with drug and exposed to hypobaric hypoxia (0.55 atmospheres) for 24 h (hypoxic group). Control mice were kept at normal atmospheric pressure (oxic group). Drugs were administered over a range of doses. The ratio of drug dose required to give an equivalent anti-tumour effect was used to determine the dose enhancement ratio. Using Fieller's theorem, 95% confidence limits were calculated. [The DER values of SR 4233 and RSU 1069 are reprinted from McAleer *et al.* (1992) with kind permission Elsevier Science.]

was also administered with an approximately isoeffective fractionated radiation regimen (5 × 3 Gy) administered over five consecutive days. The drug dose was fixed at 200 mg $kg^{-1}$ and administered 30 min before irradiation. Three different drug scheduling regimens were used: 200 mg $kg^{-1}$ on day 1, 100 mg $kg^{-1}$ on days 1 and 3 or 40 mg $kg^{-1}$ on each of the five consecutive radiation days.

### Results

*Systemic toxicity*

Following administration of AQ4N at a range of doses in oxic and hypoxic mice (Table 1) systemic toxicity was assessed using weight loss following treatment (Figure 2a). Normally oxygenated mice showed minimal weight loss when given AQ4N up to doses of 400 mg $kg^{-1}$ (0.9 mmol $kg^{-1}$), although some mice showed a small weight loss in the range 5–10%. There was no evidence of a dose–response relationship. Systemic toxicity was not increased when administration of AQ4N at low doses (up to 50 mg $kg^{-1}$) was followed by induction of hypobaric hypoxia (Figure 2a). At higher doses (100–200 mg $kg^{-1}$) there was appreciable weight loss ($P<0.001$), and therefore doses beyond 200 mg $kg^{-1}$ were not tested under hypoxic conditions.

We have also studied the systemic toxicity of the bioreductive drugs RSU 1069 and SR 4233 under oxic and hypoxic conditions (Figure 2b and c). When RSU 1069 was given under oxic conditions there was no significant weight loss with increasing drug dose up to 150 mg $kg^{-1}$. In combination with hypoxia a significant increase was found ($P<0.001$). For SR 4233 given to normally oxygenated mice there was a significant increase in weight loss up to the normally reported maximum tolerated dose of 50 mg $kg^{-1}$ ($P<0.001$). In combination with hypobaric hypoxia there was a significant

**Figure 2** Relationship between percentage weight loss post treatment and dose of bioreductive drug. The weight loss at 2–3 days post treatment is expressed as a percentage of pretreatment weight for individual mice. This is plotted against the logarithm of the drug dose for (a) AQ4N, (b) RSU 1069 and (c) SR 4233. Mice were treated over a range of drug doses both at normal atmospheric pressure (oxic, □) and immediately before induction of hypobaric hypoxia at 0.55 atmospheres for 24 h (hypoxic, ■). (The data for SR 4233 are reprinted from McAleer *et al.*, 1992.)

enhancement of systemic toxicity in combination with SR 4233, with weight losses of less than 10% only being observed at very low doses (<6 mg kg$^{-1}$).

### *Tumour response: AQ4N with hypobaric hypoxia*

The anti-tumour effect of AQ4N was assessed by comparing the dose–response curves in mice treated at 1 and 0.55 atmospheres. There was a left shift of the dose–response curve under hypoxic conditions with a DER of 5.1 (Figure 3). The values derived previously for SR 4233 and RSU 1069 were 8.8 and 8.5 respectively (McAleer *et al.*, 1992). The DER results obtained for the three drugs were not significantly different from each other. However, the 95%

**Figure 3** Relationship between tumour growth delay and dose of drug. The tumour growth delay is plotted against the logarithm of dose of AQ4N for oxic (□) and hypoxic (■) treatment groups. The parallel regression lines have been plotted; the dose enhancement ratio (DER) is the anti-log of the horizontal separation of the parallel lines.

**Figure 4** Relationship between tumour growth delay and dose of radiation. Tumour growth delay (mean ± s.e.) plotted against radiation dose administered as a single fraction.

confidence intervals for all three drugs (Table I) show that the DER values are significantly different from 1.0 ($P < 0.005$). This indicates that there is a significant increase in the cytotoxic effects of these drugs with hypoxia, and is consistent with the proposal that they undergo *in vivo* bioreductive activation.

### *Tumour response: AQ4N and single dose radiation*

With single fractions of radiation ranging from 7.5 to 25 Gy there was increasing tumour growth delay (Figure 4). For 12 Gy tumour growth delay was 6.93 (s.e. = 0.95). When AQ4N (200 mg kg$^{-1}$) was given alone the tumour growth delay was 3.09 (s.e. = 0.50). In combination these modalities (AQ4N, 200 mg kg$^{-1}$; radiation, 12 Gy) gave a tumour growth delay of approximately 18 days (Figure 5). From the radiation dose–response curve 24 Gy, as a single dose, is required to give an 18 day growth delay. Thus AQ4N reduced by 50% the radiation dose required to give an anti-tumour effect equivalent to that of radiation alone.

In order to maximise the benefit from drug–radiation interactions, it was important to identify the most effective time interval between administration of the two modalities. Initially the drug was administered at a range of times from 90 min before to 60 min after radiation treatment. All of these schedules gave an additive effect with tumour growth delays of approximately 18 days (no significant difference). The scheduling experiment was repeated twice using a wider range of time to identify the period over which the additive interaction could be obtained. The interaction was present over a long time period, with the maximal interaction being observed when the drug was administered from 4 days before

**Figure 5** Tumour growth delay when AQ4N was administered before or after a single dose of radiation (12 Gy). AQ4N (200 mg $kg^{-1}$) was administered at a range of times up to 120 h before and 48 h after a single dose of X-irradiation (12 Gy). Tumour growth delay (mean ± s.e.) (6–18 animals per group) is plotted against time of administration. Bars show the tumour growth delay obtained for AQ4N and radiation administered alone (mean ± s.e.). The results are the pooled data from three experiments.

**Figure 6** Tumour growth delay when AQ4N was administered in combination with fractionated radiation (5 × 3 Gy). AQ4N (200 mg $kg^{-1}$) was administered 30 min before radiation. The drug was given as a single dose on the first day of treatment, or in multiple doses as indicated. A single dose of drug (200 mg $kg^{-1}$) combined with fractionated radiation was significantly different from fractionated radiation alone ($P<0.01$). Multiple doses of drug (5 × 40 mg $kg^{-1}$) with fractionated radiation was significantly more effective than a single dose of drug when combined with fractionated radiation ($P<0.01$). The results of single-dose radiation (12 Gy) in combination with AQ4N (200 mg $kg^{-1}$) are included for comparison.

to 6 h after the radiation treatment (Figure 5). An appreciable, but diminishing, effect was still seen when the drug was administered up to 48 h after radiation.

*Tumour response: AQ4N with fractionated radiation*

In clinical practice, radiotherapy is given as a fractionated regimen. Therefore, AQ4N was assessed for its anti-tumour efficacy when administered with 15 Gy given in five daily fractions of 3 Gy (Figure 6). Drug was administered in all schedules 30 min before irradiation. When given as a single dose (1 × 200 mg $kg^{-1}$) AQ4N gave a marked enhancement of anti-tumour effect as compared with either modality alone ($P<0.01$). AQ4N given in two doses (2 × 100 mg $kg^{-1}$) slightly improved the outcome. When AQ4N was given on each day of radiation treatment, but with the same total dose (i.e. 5 × 40 mg $kg^{-1}$), the anti-tumour effect was significantly increased as compared with the single-dose AQ4N with a fractionated radiation regimen ($P<0.01$) (Figure 6). This combination was as effective as that obtained when single doses of both modalities were combined.

### Discussion

AQ4N is a novel alkylaminoanthraquinone N-oxide which shows minimal cytotoxicity to cells even at high concentrations (Patterson *et al.*, 1995). On exposure to a hypoxic cellular environment the major metabolite formed is AQ4; this is a stable, cytotoxic compound with high affinity for DNA. The hypoxic cytotoxicity of AQ4N to V79 cells is twice that found under normal oxygenation. This can be increased to 100 times when rat liver microsomes are present, suggesting that reductive metabolism of AQ4N requires the presence of specific microsomal enzymes (Patterson, 1993). It is known that metabolism of AQ4N involves cytochrome P450 (Graham *et al.*, 1993), which is normally down-regulated *in vitro* (Krupski *et al.*, 1985). This could explain why only limited reduction of AQ4N is found *in vitro*. We have recently shown that excised T50/80 tumour cells can metabolise AQ4N under hypoxic conditions but that this ability is lost in isolated cells within 24 h (unpublished data).

Several advantages result from the differences in the chemical properties of AQ4N and its reduction product AQ4. Firstly, the stability of AQ4 and its high binding affinity for DNA allows for a long residence time in the cells in which it is formed. The demonstrated long interval of interaction between drug and radiation (Figure 5) suggests that this can occur *in vivo*. It is also consistent with the evidence that mitoxantrone, a close analogue of AQ4, has a long elimination half-life *in vivo* of several hours to days (reviewed by Faulds *et al.*, 1991). In contrast, the elimination half-life of AQ4N in mice following i.p. administration is approximately 30 min (LH Patterson and MA Graham, unpublished results); this is consistent with the low affinity of AQ4N for DNA. Secondly, when AQ4 is bound to DNA, it acts as a topoisomerase II inhibitor (Patterson, 1993; PJ Smith, personal communication). Thus, AQ4 should be cytotoxic to the cell in which it is formed, even if that cell later becomes reoxygenated and attempts to divide. Finally, any diffusion of AQ4, the reduction product, to an adjacent cell would result in toxicity to that cell irrespective of its level of oxygenation.

In previous studies we have derived a dose enhancement ratio (DER) to assess bioreductive activation of drugs *in vivo*. This was measured by assessing the enhancement of the anti-tumour effect of the drug when the tumour was rendered hypoxic *in vivo* using hypobaric hypoxia (McAleer *et al.*, 1992). When AQ4N was tested, it showed limited anti-tumour effect at normal levels of oxygenation. With hypoxia a significantly lower dose of AQ4N was required to give the same anti-tumour effect with a DER of 5.1 (Figure 3), suggesting that AQ4N may be toxic to hypoxic cells *in vivo* through the production of AQ4. Although the DER for AQ4N was less than that obtained previously with SR 4233 and RSU 1069 (Table I), AQ4N showed significant bioreductive potential as measured by this test system.

Ideally a bioreductive drug should show selective toxicity to the treatment-resistant hypoxic cells of tumours, without toxicity to normally oxygenated tissues. This should result in sparing of normal tissues and yield a high therapeutic ratio. In mice kept at normal levels of oxygenation AQ4N showed only a minimal increase in systemic toxicity (as measured by weight loss) with doses that showed measurable anti-tumour effect. In particular, AQ4N showed almost no toxicity at doses that gave effective enhancement of radiation induced cell kill. In addition, systemic toxicity was not enhanced by hypoxia at doses up to 50 mg $kg^{-1}$, although hypoxia did potentiate systemic toxicity at higher doses of AQ4N. This suggests that normal tissues in these mice could metabolise the drug only when oxygen levels were artificially reduced.

The striking toxicity of SR 4233 in oxic mice at doses approaching the maximum tolerated dose suggest that sufficient metabolism of SR 4233 occurred systemically under conditions of normal oxygenation. This effect was further enhanced by hypoxia. Minchinton and Brown (1992)

obtained similar results for SR 4233 when it was administered in combination with normobaric hypoxia (10% oxygen). In contrast, RSU 1069 showed significant toxicity only in hypoxic mice. The recent study by Koch (1993) provides an explanation for the higher systemic toxicity of SR 4233 shown both in our studies and that of Minchinton and Brown (1992).

Clinical use of bioreductive drugs will require their combination with an agent toxic to well-oxygenated cells. Investigation of AQ4N with radiation showed it to be a very efficient dose-sparing agent, giving a substantial reduction (50%) in radiation dose to give the same anti-tumour effect (Figure 5). Several similar studies have shown additive or supra-additive interactions when bioreductive drugs are combined with radiation treatments (Brown and Lemmon, 1991; Cole *et al.*, 1991; Grau and Overgaard, 1991). The maximal effect reported in these studies was found when the interval between administration of the two modalities was less than 24 h. With AQ4N a maximal effect can be elicited even if the drug is administered 4 days before radiation. [The current experiments did not lend themselves to full isobologram analysis since AQ4N alone does not give an 18 day growth delay (unpublished data), thus preventing the derivation of the complete additivity envelope].

If radiation is administered at approximately the same time as a putative bioreductive drug, a positive interaction can be anticipated since the two modalities are cytotoxic to different cell subpopulations, i.e. the oxic and hypoxic fractions. With AQ4N the observed interaction is maintained even when the drug is administered up to 4 days before radiation. As discussed, the major reduction product of AQ4N *in vitro* is the highly DNA affinic agent AQ4. Our results suggest that AQ4N ($t_{1/2}$ = 30 min in mice) is metabolised to a cytotoxic agent, presumably AQ4, with a long half-life *in vivo*. Even if the drug is administered 4 days before radiation almost no effect is seen on tumour growth, yet the additional insult of irradiation causes a much greater anti-tumour effect than that found in controls. Administration of radiation to a tumour results in oxic cell kill and reoxygenation of hypoxic cells, which may divide to repopulate the tumour. If the hypoxic cells contain bound AQ4 this will inhibit cell cycle progression by reoxygenated cells since it is a topoisomerase II inhibitor. Our results suggest that this can occur even 4 days after administration of the pro-drug. On a longer time scale AQ4 containing hypoxic cells should die and be lost from the tumour. New hypoxic cells would be generated and the interaction with radiation lost. We were unable to demonstrate this as the end point of tumour growth delay restricts the interval between treatments which can be assessed.

When AQ4N was administered up to 6 h after irradiation the maximal growth delay of 18 days was elicited. Since the enhanced anti-tumour effect occurs post irradiation, this provides evidence that AQ4N is not a radiosensitiser but is acting as a bioreductive drug. There was a 6–48 h period after irradiation when the enhanced anti-tumour effect was still apparent although diminishing. If AQ4N was given immediately after irradiation it may be metabolised to produce AQ4 in hypoxic cells and prevent them from repopulating the tumour. If this time interval is prolonged regeneration of the oxic fraction will occur from the residual, mainly hypoxic, cells. Thus the number of cells sensitive to AQ4N will be reduced. Moore (1988) used split dose data and found that the oxic fraction was regenerated by about 3 days. This would explain why AQ4N retained its activity when administered up to 48 h after irradiation as the tumour would still have a large number of sensitive hypoxic cells.

Clinical radiotherapy is normally given as a fractionated regimen. When AQ4N was administered with fractionated radiation the extent of anti-tumour toxicity was similar to that obtained with the single-dose experiments. The most effective outcome was obtained when the AQ4N was also split into five equal doses given daily with radiation. This suggests that on each day of administration a small additional fraction of acutely hypoxic cells was killed, increasing the anti-tumour effect. The phenomenon of acute hypoxia has been described previously by Trotter *et al.* (1990). A significantly increased anti-tumour effect was obtained with a single dose of drug combined with five fractions of radiation, although this was not as effective as that found when both modalities were administered as fractionated regimens. The results suggest that AQ4N may have considerable potential for combination with fractionated radiation schedules at drug doses that should not cause systemic toxicity. Further studies are planned to examine dosing schedules for both modalities.

In conclusion, AQ4N is a novel drug which shows bioreductive activation *in vivo*. We have shown AQ4N to have an additive anti-tumour effect when combined with radiation, even when there is a long separation time between administration of the two modalities. Our studies have highlighted four major properties of AQ4N which might be exploited in clinical studies. (1) When given in combination with radiation AQ4N allows a significant reduction in radiation dose for an equivalent anti-tumour effect. (2) AQ4N has minimal toxicity at doses effective in combined modality experiments, suggesting that the radiation sparing may be achieved without adverse systemic toxicity. (3) The dose-sparing effect can be elicited even if AQ4N is administered several hours (even days) before the radiation. This scheduling should allow sufficient time for the prodrug to be eliminated from normal tissues before irradiation, thus reducing the risk of enhanced, drug-related, normal tissue toxicity in the radiation field. (4) An equally effective interaction is obtained when AQ4N is combined with fractionated radiation, suggesting potential for AQ4N to be combined with clinical radiotherapy regimens.

**Acknowledgements**

The technical assistance of Kevin McAdam, Dawn Kirk, Ketan Ruparelia and Peter Gray is gratefully appreciated. Drugs were provided by Dr VL Narayanan, Drug Synthesis and Chemistry Branch, Division of Cancer Treatment, NCI, Bethesda, Maryland (SR 4233), and Professor GE Adams, Drug Synthesis and Evaluation Group, MRC Radiobiology Unit, Chilton, UK (RSU 1069). This work has been supported by the Ulster Cancer Foundation, N. Ireland, Cancer Research Campaign, UK, and the British Technology Group plc UK.

### References

ARMITAGE P AND BERRY G. (1987). *Statistical Methods in Medical Research*, 2nd ed, pp. 484–498, Blackwell Scientific Publications: Oxford.

BREMNER JCM, STRATFORD IJ, BOWLER J AND ADAMS GE. (1990). Bioreductive drugs and the selective induction of tumour hypoxia. *Br. J. Cancer*, **61**, 717–721.

BROWN JM AND LEMMON MJ. (1991). Tumour hypoxia can be exploited to preferentially sensitize tumours to fractionated irradiation. *Int. J. Radiat. Oncol. Biol. Phys.*, **20**, 457–461.

BUSH RS, JENKIN RDT, ALLT WEC, BEALE FA, BEAN H, DENBO AJ AND PRINGLE JF. (1978). Definite evidence for hypoxic cells influencing cure in cancer therapy. *Br. J. Cancer*, **37** (Suppl. III), 302–306.

COLE S, STRATFORD IJ, BOWLER J, NOLAN J, WRIGHT EG, LORIMORE SA AND ADAMS GE. (1991). Oral (p.o.) dosing with RSU 1069 or RB 6145 maintains their potency as hypoxic cell radiosensitizers and cytotoxins but reduces systemic toxicity compared with parenteral (i.p.) administration in mice. *Int. J. Radiat. Oncol. Biol. Phys.*, **21**, 387–395.

DENNY WA AND WAKELIN LPG. (1990). Kinetics of binding of mitoxantrone, ametantrone and analogues to DNA: relationship with binding mode and anti-tumour activity. *Anti-Cancer Drug Design*, **5**, 189–200.

FAULDS D, BALFOUR JA, CHRISP P AND LANGTRY HD. (1991). Mitoxantrone. A review of its pharmacodynamic and pharmacokinetic properties and therapeutic potential in the chemotherapy of cancer. *Drugs*, **41**, 400–409.

GRAHAM MA, KING LH, WORKMAN P, HENDERSON C, WOLF CR AND PATTERSON LH. (1993). Identification of cyt P450 2C8 as the major human P450 isoform involved in the bioreduction of the novel anthraquinone di-N-oxide, AQ4N. *Br. J. Cancer*, **67**, (Suppl. XX), 9.

GRAU C AND OVERGAARD J. (1991). Radiosensitizing and cytotoxic properties of mitomycin C in a C3H mouse mammary carcinoma *in vivo*. *Int. J. Radiat. Oncol. Biol. Phys.*, **20**, 265–269.

KOCH CJ. (1993). Unusual oxygen concentration dependence of toxicity of SR-4233, a hypoxic cell toxin. *Cancer Res.* **53**, 3992–3997.

KRUPSKI G, KIEFER F AND WEIBEL FJ. (1985). Variability in the expression of xenobiotic metabolising enzymes during the growth cycle of rat hepatoma cells. *Xenobiotica*, **15**, 781–787.

McALEER JJA, McKEOWN SR, MACMANUS MP, LAPPIN TRJ AND BRIDGES JM. (1992). Hypobaric hypoxia: a method for testing bioreductive drugs *in vivo*. *Int. J. Radiat. Oncol. Biol. Phys.*, **23**, 551–555.

MINCHINTON AI AND BROWN JM. (1992). Enhancement of the cytotoxicity of SR 4233 to normal and malignant tissues by hypoxic breathing. *Br. J. Cancer*, **66**, 1053–1058.

MOORE JV. (1988). The dynamics of tumour cords in an irradiated mouse mammary carcinoma with a large hypoxic cell component. *Jpn J. Cancer Res. (Gann)*, **79**, 236–243.

PATTERSON LH. (1989). Anthraquinone anticancer compounds with (disubstituted amino-N-oxide)alkylamino substituent. UK Patent GB 2 237 283.

PATTERSON LH. (1993). Rationale for the use of aliphatic N-oxides of cytotoxic anthraquinones as prodrug DNA binding agents: a new class of bioreductive agent. *Cancer Metast. Rev.* **12**, 119–134.

PATTERSON LH, CRAVEN MR, FISHER GR AND TEESDALE-SPITTLE P. (1995). Aliphatic amine N-oxides of DNA binding agents as bioreductive drugs. *Oncol Res.* (in press).

TROTTER MJ, CHAPLIN DJ AND OLIVE PL. (1990). Possible mechanisms for intermittent blood flow in the murine SCCVII carcinoma. *Int. J. Radiat. Biol.*, **60**, 139–146.

WORKMAN P. (1992). Design of novel bioreductive drugs. In: Workman P. (ed.) *New approaches in Cancer Pharmacology: Drug Design and Development*, pp. 63–74. Springer: Berlin.

WORKMAN P AND STRATFORD IJ. (1993). The experimental development of bioreductive drugs and their role in cancer therapy. *Cancer Metast Rev*, **12**, 73–82.